# Assessing the Risks of Pesticide Exposure: Implications for Endocrine Disruption and Male Fertility

**DOI:** 10.3390/ijms25136945

**Published:** 2024-06-25

**Authors:** Claudine Uwamahoro, Jae-Hwan Jo, Seung-Ik Jang, Eun-Ju Jung, Woo-Jin Lee, Jeong-Won Bae, Woo-Sung Kwon

**Affiliations:** 1 Department of Animal Science and Biotechnology, Kyungpook National University, Sangju 37224, Republic of Korea; claudineuwa20@gmail.com (C.U.); ocjallk@naver.com (J.-H.J.); todwnl5787@naver.com (S.-I.J.); red0787@naver.com (E.-J.J.); wj9059lee@naver.com (W.-J.L.); jwbae1822@gmail.com (J.-W.B.); 2 Research Institute for Innovative Animal Science, Kyungpook National University, Sangju 37224, Republic of Korea

**Keywords:** pesticides, sperm capacitation, male infertility, endocrine system dysfunction

## Abstract

Pesticides serve as essential tools in agriculture and public health, aiding in pest control and disease management. However, their widespread use has prompted concerns regarding their adverse effects on humans and animals. This review offers a comprehensive examination of the toxicity profile of pesticides, focusing on their detrimental impacts on the nervous, hepatic, cardiac, and pulmonary systems, and their impact on reproductive functions. Additionally, it discusses how pesticides mimic hormones, thereby inducing dysfunction in the endocrine system. Pesticides disrupt the endocrine system, leading to neurological impairments, hepatocellular abnormalities, cardiac dysfunction, and respiratory issues. Furthermore, they also exert adverse effects on reproductive organs, disrupting hormone levels and causing reproductive dysfunction. Mechanistically, pesticides interfere with neurotransmitter function, enzyme activity, and hormone regulation. This review highlights the effects of pesticides on male reproduction, particularly sperm capacitation, the process wherein ejaculated sperm undergo physiological changes within the female reproductive tract, acquiring the ability to fertilize an oocyte. Pesticides have been reported to inhibit the morphological changes crucial for sperm capacitation, resulting in poor sperm capacitation and eventual male infertility. Understanding the toxic effects of pesticides is crucial for mitigating their impact on human and animal health, and in guiding future research endeavors.

## 1. Introduction

The yields of crops intended for human consumption face a significant risk of loss due to encounters with various pests [1,2]. Studies estimate that approximately 50% to over 80% of crops are lost due to exposure to pests [2,3]. Consequently, pest and disease control have proven beneficial in increasing global crop production for several years [1,2,4]. Similarly, the prevalence of malaria has been increasing in different parts of the world [5]. Reports indicate that in tropical countries, malaria’s prevalence among pregnant women ranges between 10% and 20%, whereas among children, it varies from 4.7% to 49.7%. Therefore, the World Health Organization advocates for the use of insecticide-treated nets to combat malaria [6,7]. Pesticides such as herbicides, insecticides, fungicides, and rodenticides have emerged as a solution in both agriculture and the health sector [1,8]. A variety of pesticides, including pyrethroids, organophosphates, phenylpyrazole, benzoylphenylurea, methylenedioxyphenyl, and organochlorines, are available on the market and are utilized to target various types of pests, including weeds, pathogens, and animal pests, as well as for the treatment of conditions such as scabies and diseases related to lice and mites [3,7,8]. Research indicates a per capita increase in the use of pesticides in South America, especially in Brazil, from 1985 to 2015, during which Brazil experienced a substantial rise in pesticide consumption, with usage levels escalating by 700% over this 30-year period [9,10]. In different regions of Africa, a variety of pesticides are commonly employed for pest management, not only in agriculture but also for controlling the population of tsetse flies, houseflies, mosquitoes, and ticks [8,11]. Furthermore, several Asian countries use pesticides for agriculture. For instance, Kazakhstan, being a major wheat-producing country, grapples with pest, pathogen, and weed infestations, resulting in crop loss. Therefore, pesticides are utilized to safeguard crops against these detrimental factors. India stands out as the largest consumer of pesticides globally [1,12]. The extensive utilization of pesticides in both agriculture and the healthcare sector has resulted in the direct or indirect exposure of humans and animals to pesticides in their daily routines [1,13,14]. Although pesticides play a crucial role in controlling pests and boosting agricultural production [15], research underscores the adverse effects of pesticides on humans and animals owing to their toxicity [13,16,17]. Notably, although pesticides are designed to target specific organisms, there remains a high risk of unintentionally affecting non-targeted organisms [18]. Exposure to pesticides can pose direct health risks for humans, including headaches, nausea, and eye and skin irritation [13,14,19]. Furthermore, both unintentional and deliberate contact with pesticides can result in acute and/or chronic toxicity [19,20,21]. Furthermore, the presence of organophosphate compounds such as profenofos and malathion exacerbates the cytotoxic effects on organs such as the liver, heart, kidneys, and muscles [7,16,17,22]. Previous studies report that chlorpyrifos (CPF) induces hepatotoxicity in rats by altering liver marker enzymes such as ALP (alkaline phosphatase) enzyme commission number (EC) 3.2.3.1, AST (aspartate aminotransferase) EC 2.6.1.1, and LDH (lactase dehydrogenase) EC 1.1.1.27, while also causing histopathological modifications in the liver [23,24]. Additionally, lufenuron, a benzoylphenylurea insecticide, causes cardiac toxicity in fish by inducing histopathological abnormalities such as congestion, edema, myofibrosis, and neutrophilic myocarditis [17,24]. Pesticides not only affect the liver, kidneys, and heart, but also have adverse impacts on reproduction, potentially leading to infertility. Research indicates that pesticides such as novaluron, lufenuron, temephos, fipronil, and bifenthrin contribute to male infertility by reducing sperm capacitation, sperm motility, motion parameters, and sperm cell viability [25,26,27,28,29]. After ejaculation, when the sperm enter the female reproductive tract, they must undergo a physiological process to gain the ability to fertilize an egg. This process is known as sperm capacitation [30,31]. Furthermore, pesticides can influence sperm function by triggering abnormal molecular changes such as protein kinase A (PKA) activity, tyrosine phosphorylation, and the phosphoinositide 3-kinases/protein kinase B (PI3K/AKT) signaling pathways [32,33]. Moreover, flufenoxuron exerts a toxic impact on embryos and the placenta during early pregnancy in sows [33,34]. While numerous studies have documented the adverse effects of pesticides on various organs, there remains a need for a detailed review focusing specifically on molecular mechanisms by which pesticides impact male fertility. In this review, we highlight the toxic effects of pesticides on both humans and animals, shedding light on male fertility, as well as the mechanisms associated with such effects. Furthermore, we identify knowledge gaps that warrant further studies.

## 2. General Toxicity of Pesticides

### 2.1. Overall Toxicity

The endocrine system comprises a network of glands that produce and secrete hormones, which regulate various physiological functions in the body. This intricate system can be disrupted by a range of chemicals known as endocrine-disrupting chemicals (EDCs). These substances have the potential to alter the normal functioning of the endocrine system in humans and animals, thereby posing significant health risks [35,36]. Pesticides act as EDCs by mimicking the regular functions of the endocrine system, consequently disrupting the normal operation of various organs in the body [37]. Studies have demonstrated that exposure to pesticides can result in eye irritation, dermatitis, and acute diseases such as cardiovascular issues, neurological damage, respiratory issues, birth defects, cancer, kidney and liver disorders, and even mortality [38]. Moreover, the oral intake of CPF and permethrin may lead to symptoms such as diarrhea, abdominal pain, vomiting, loss of consciousness, headaches, sore throats, epidermal lesions, and gastrointestinal mucosal irritation in humans and animals [20,39]. Furthermore, pesticides have adverse effects on reproductive organs in both humans and animals, resulting in diminished fertility [40]. Overall, pesticide toxicity encompasses a range of potential harms to humans and animals. In this review, we mainly discuss how pesticides induce neurological damage, hepatotoxicity, cardiotoxicity, pulmonary toxicity, and reproductive toxicity.

### 2.2. Neurotoxicity of Pesticides

Exposure to pesticides can disrupt brain function by affecting thyroid hormone levels. Thyroid hormones, such as thyroxine (T4) and triiodothyronine (T3), are vital for regulating metabolism, energy production, and the function of the cardiovascular and nervous systems [41,42,43]. Chlorinated hydrocarbons like Dichloro-Diphenyl-Trichloroethane (DDT), for instance, can alter thyroid hormone levels, potentially leading to hypothyroidism, which can cause brain dysfunction, manifesting as symptoms such as sleepiness, paranoia, and depression [42,44]. Dieldrin, another organochloride pesticide, has been linked to hypothyroidism in women as well [42,44,45]. Moreover, acetylcholinesterase (AChE) EC 3.1.1.7, a crucial enzyme involved in neurotransmission, facilitates the degradation of the neurotransmitter acetylcholine through enzymatic hydrolysis [46,47,48,49]. Studies indicate that AChE can be inhibited by organophosphate pesticides, resulting in elevated levels of in acetylcholine within neuronal synapses where the neurotransmitter is released in mammals [46,47,49]. Organophosphates donate their phosphate group to the serine residue of AChE, forming a stable phosphorylated complex [20,47,49]. This results in the inactivation of AChE, preventing it from efficiently breaking down acetylcholine and disrupting normal neurotransmission [49,50]. Consequently, organophosphates can induce elevated synaptic acetylcholine levels, leading to the massive release of glutamine, which, in turn, sustains and maintains status epilepticus [51,52]. For instance, monocrotophos poisoning rapidly inhibits AChE in various brain regions, particularly the striatum and hippocampus [53]. This indicates that AChE inhibition plays a significant role in the neurotoxic effects observed in these areas [53]. In addition to organophosphates, benzoyl phenylurea and deltamethrin insecticides can also inhibit AChE [54,55,56]. In addition, allethrin decreases cell viability in human dopaminergic neuroblastoma SH-SY5Y cells by significantly elevating ROS levels. This increase in reactive oxygen species (ROS) leads to oxidative stress, which contributes to cellular damage and impairs the overall health and function of these neuronal cells (Table 1). However, few studies have examined the effects of these pesticides on mammals. Furthermore, the administration of CPF has been shown to result in a substantial decrease in the activity of AChE, butyrylcholinesterase (BChE) EC 3.1.1.8, and carboxylesterase (CbE) EC 3.1.1.1, accompanied by a markedly elevated level of deoxyribonucleic acid (DNA) damage. These findings indicate a significant impact on neurochemical processes and genetic integrity, thus highlighting the intricate and deleterious effects of CPF exposure on both enzymatic activities and genomic stability [57,58]. Synthetic pyrethroids, including cyhalothrin and deltamethrin, have been demonstrated to decrease acetylcholine levels in the hippocampus of rats, whereas allethrin has the opposite effect [59,60]. In contrast, cypermethrin, also a synthetic pyrethroid, exerts a detrimental effect on the nervous system by inducing abnormal neuronal discharge [61]. Specifically, cypermethrin alters the behavior of voltage-dependent transient outward potassium (K^+^) currents (I_K_) and delayed rectifier potassium (K^+^) currents (I_A_) in neurons in a concentration-dependent manner, with higher concentrations (10^−7^ M) significantly impacting their properties. These changes in I_K_ and I_A_ kinetics could result in neurons experiencing prolonged periods of depolarization, delaying action potential generation, broadening the duration of action potentials, and boosting the frequency of repetitive firing [62]. Furthermore, cyfluthrin has been found to stimulate hippocampal inflammation and ATPase in hippocampal neurons in a dose-dependent manner. This is evidenced by elevated levels of tumor necrosis factor-alpha (TNF-α) and interleukin-6 (IL-6) in Wistar rats exposed to cyfluthrin [63]. TNF-α possesses the capability to induce nerve damage by promoting neuroinflammation and encouraging T cells to release inflammatory factors [64,65]. In contrast, IL-6 exhibits a wide range of biological functions. Specifically, it plays a role in regulating the immune system’s response and facilitating the differentiation of B cell precursors into antibody-producing cells. This function is crucial for reducing inflammation and preventing neuronal degeneration and necrosis [64,66]. In summary, the evidence presented underscores the significant impact of pesticide exposure on neurological function, including alterations in neurotransmission, enzymatic activity, and immune responses.

### 2.3. Cardiotoxicity of Pesticides

Numerous studies have highlighted the adverse impact of pesticides on cardiac health, often resulting in cardiac abnormalities and, in severe cases, death [41,67]. Organophosphate pesticides, such as CPF, methyl parathion, and dichlorvos, have been linked to various cardiovascular complications, including a transient increase in sympathetic tone, a prolonged period of parasympathetic activity, and electrocardiography (ECG) abnormalities such as QT prolongation, leading to torsade de pointes (TdP) and myocardial damage [67,68]. Additionally, CPF has been associated with the inhibition of heart cholinesterase and the down-regulation of muscarinic receptors [69]. In another study, exposure to diazinon, propoxur, and CPF induced cardiotoxicity in rabbits [70]. Specifically, these pesticides led to the thinning of left ventricular (LV) walls, reduced myocardial mass, and impaired systolic and diastolic performance. Furthermore, exposure to both diazinon and propoxur resulted in the identification of fibrosis and hemorrhage in heart muscle tissue [70]. Moreover, organophosphate pesticides and carbamates have been found to cause sinus tachycardia (elevated heart rhythm) and sinus bradycardia (slower-than-normal heart rate) [41,68]. Furthermore, studies have demonstrated that pesticides can cause ST-T changes, indicating various cardiac conditions such as myocardial infarction and myocardial ischemia [19,41]. This group of pesticides primarily functions by inhibiting the enzyme AChE, preventing the breakdown of acetylcholine and leading to its accumulation in the body. Consequently, this excess acetylcholine activates cholinergic receptors, specifically nicotinic and muscarinic receptors, in various cells [71]. Furthermore, studies have reported that perinatal exposure to dichlorodiphenyltrichloroethane (DDT) results in increased blood pressure by over-activating the renin–angiotensin system (RAS). This prompts the kidneys to upregulate sodium (Na+) transporters, promoting Na+ retention and elevating blood pressure. Aldosterone plays a pivotal role in this process. Thus, DDT exposure contributes to hypertension by altering kidney function and also leads to cardiac hypertrophy [72]. Moreover, a recent study found that dichlorvos, also known as 2,2-dichlorovinyl dimethyl phosphate (DDVP), induces necrotic cell death in H9C2 cells by triggering endoplasmic reticulum (ER) stress. However, H9C2 cells are protected from DDVP-induced toxicity by SIRT1, which enhances autophagy. This, in turn, mitigates the generation of reactive ROS and ER stress, subsequently inhibiting induced necroptosis [71]. Moreover, exposure to low doses of permethrin insecticide during early life results in lasting effects, including cardiac hypotrophy and heightened calcium (Ca^2+^) and Nrf2 gene expression levels in old age [73]. Overall, various pesticides trigger cardiac toxicity by altering heart function, ultimately leading to death.

### 2.4. Hepatotoxicity of Pesticides

Hepatotoxicity refers to the potential of a substance, such as a drug or chemical, to cause damage to the liver, resulting in liver dysfunction or injury. The liver plays an essential role in the detoxification process; however, it can be damaged by various chemicals owing to their excessive toxicity [23]. Pesticides are recognized as chemicals capable of enhancing hepatotoxicity [74]. CPF, an organophosphate pesticide, has been reported to induce oxidative stress and generate ROS in liver tissues [75]. Additionally, organophosphates such as CPF can induce liver damage by altering the levels of liver marker enzymes such as ALP, AST, and LDH, while also inducing histopathological changes in liver tissue [23]. Studies have shown that CPF increases the levels of ALP, AST, and LDP enzymes while reducing total protein and albumin in rats and mice [23,76]. Moreover, DDT, permethrin, and their combination have been found to induce cell death in hepatocytes, with the severity of necrosis increasing in a dose-dependent manner [77,78]. These pesticides not only induce cell death in hepatocytes but also reduce the concentration of α-ketoglutarate, a metabolite crucial for cellular function. This decrease is attributed to the inhibition of alanine aminotransferase (ALT) and AST enzymes, which normally produce α-ketoglutarate in healthy liver cells [77,78]. Another study reported that exposure to a high dose of DDT caused the development of hepatocellular adenomas and carcinomas [78]. Additionally, deltamethrin causes oxidative stress, resulting in significant histological, biochemical, and physiological changes in the kidneys and liver. Moreover, it leads to congestion and the widening of portal blood vessels, the infiltration of inflammatory cells between hepatic cords, and thickening of the walls of hepatic blood vessels, ultimately compromising the overall function of those organs [79]. Rajawat et al. reported that exposure to cyfluthrin resulted in increased liver weight, which might be associated with hepatic hypertrophy and hydropic degeneration, accompanied by a marked decrease in glycogen levels and a substantial increase in liver cholesterol content. Additionally, a significant elevation in ALP activity was prominently observed [80]. Furthermore, Rajawat et al. reported elevated levels of AST or SGOT (serum glutamate–oxaloacetate transaminase) EC 2.6.1.1, ALT or SGPT (serum glutamate–pyruvate transaminase) EC 2.6.1.2, and ALP in serum, indicating liver damage or injury caused by cyfluthrin [80,81]. These enzymatic elevations serve as markers of hepatocellular injury, indicating the detrimental effects of cyfluthrin on liver function [80,81]. Thus, pesticide exposure has detrimental effects on the liver, characterized by both direct toxicity to hepatocytes, leading to cell death, and the disruption of metabolic processes. This dual impact reflects the hepatotoxicity of pesticides, compromising liver health and function.

### 2.5. Pulmonary Toxicity of Pesticides

Pulmonary toxicity refers to damage or impairment to the lungs resulting from exposure to certain hazardous substances. This damage can adversely affect lung function and potentially lead to respiratory diseases. Pesticides are recognized as one category of hazardous chemicals known to cause pulmonary toxicity [82]. For instance, alpha-cypermethrin has been implicated in inducing pulmonary fibrosis by elevating hydroxyproline (Hyp) levels, alongside increased oxidative damage to pulmonary lipids and heightened inflammation, as indicated by cytokine levels in male rats [83,84]. Additionally, another study demonstrated that exposure to cypermethrin significantly enhanced pulmonary hyperplasia and necrosis, as evidenced by the increased size and abundance of alveolar cells [84]. Despite these findings, the precise molecular mechanisms underlying cypermethrin-induced lung damage require further investigation [83]. Further research investigating the impact of malathion and parathion, along with their metabolites malaoxon and paraoxon, on normal human bronchial epithelial cells (NHBECs) and small airway epithelial cells (SAECs) has revealed that while the pesticides themselves do not induce cellular death, their metabolites notably trigger cellular toxicity [85]. Moreover, Toll-like receptors (TLRs) a group of signaling receptors, are crucial in regulating inflammation and the innate immune response in the lungs and other tissues. TLR4 and TLR9, in particular, identify bacterial lipopolysaccharide (LPS) and cytosine–phosphate–guanine (CpG), respectively [86,87]. A previous study found that the intranasal administration of fipronil induces lung inflammation by upregulating the elevation of the levels of TLR4 and TLR9 in airway epithelial and vascular endothelial cells [88]. Additionally, an increase in the number of septal cells expressing TLR4 was observed [88]. Conversely, fipronil, in combination with LPS, elicits lung inflammation by activating the PCP/Wnt pathway in albino mice [89]. Wnt signaling plays an active role in the interaction between innate and adaptive immunity. Wnt proteins such as Wnt-6 trigger the PCP pathway by binding to receptors on the cell membrane [90]. Additionally, the downstream regulation of target genes is facilitated by the collaboration of the PCP pathway with Jnk signaling. MAPK8, also known as Jnk-1 and a member of the Jnk family of kinases, is activated via Wnt/PCP receptor signaling when cells are exposed to inflammation, oxidative stress, DNA damage, osmotic stress, infection, or cytoskeletal changes. Research demonstrates that a low dose (4.75 mg/kg) of fipronil, with or without LPS, activates Mitogen-activated protein kinase (MAKP8) via Wnt/PCP signaling [89]. Furthermore, exposure to methoxychlor, parathion, and piperonyl butoxide exacerbates allergic airway inflammation in mice [91]. In the context of allergic airway inflammation, eosinophils play a crucial role by releasing inflammatory mediators such as KC, RANTES, and MIP-1, which can induce tissue damage and worsen airway obstruction [91]. Exposure to methoxychlor, parathion, and piperonylbutoxide leads to increased eosinophil counts and chemokine levels, thereby impacting the severity of inflammation in mice [91]. Furthermore, T-lymphocytes, particularly CD4+ T cells expressing Th2 cytokines such as IL-4, -5, -6, and -13, contribute to eosinophil activation and recruitment [91,92]. Exposure to immunosuppressive pesticides results in a dose-dependent increase in Th2, alongside Th1 cytokines such as IFN and TNF, potentially exacerbating allergic airway inflammation [91]. In summary, pesticides pose a significant risk to lung health, causing respiratory irritation, exacerbating existing conditions and potentially leading to more severe respiratory effects.

**Table 1:** General toxicity of pesticides.

Organ	Pesticide, Dose	Effect	Species	Reference
Pulmonary toxicity	Cypermethrin (0.5%) in a time-dependent manner	An increase in the number of alveolar cells was observed following 10 d of cypermethrin exposure.	*Albino mice*	[84]
	Alpha-cypermethrin, 14.5 mg/kg; fipronil, 40 mg/kg-BW	Inflammation of the lungs, leading to pulmonary edema, alveolitis, and pulmonary fibrosis, as well as an increase in lung weight across all treatment groups.	*Albino rats*, *Wistar rats*	[83,93]
	Methoxychlor and piperonyl butoxide, 30 or 300 mg/kg/day; parathion, 0.15 or 1.5 mg/kg/day	Caused allergic airway inflammation	*NC/Nga mice*	[91]
Hepatotoxicity	Deltamethrin, 300 mg/kg diet, deltamethrin, 1.28 mg/kg-BW	Deltamethrin induced oxidative stress, leading to histopathological, biochemical, and physiological alterations in the kidney and liver. In addition, it congested and widened portal blood vessels, inflammatory cells between hepatic cords, and thickened the walls of hepatic blood vessels.	*Cobb broiler chicks*, *Sprague Dawley rats*	[79,94]
	Chlorpyrifos, 5.4 mg/kg-BW	Elevated levels of serum enzymes, including ALP, ALT, AST, and LDH, following exposure.	*Wistar rats*	[23]
	Triflumuron, 350 mg/kg-BW	Induced oxidative stress and reactive oxygen species (ROS) generation in liver tissues.	*BalbC mice*	[76]
	DDT, 500 ppm	Hepatocellular adenomas and carcinomas	*F344 rats*	[78]
Neurotoxicity	Deltamethrin, 7.2 mg/kg-BW	Decreased AChE activity, leading to brain dysfunction.	*Wistar rats*	[56]
	Cyhalothrin and deltamethrin, 60 mg/kg	Cyhalothrin and deltamethrin decreased acetylcholine levels in the hippocampus of rats, whereas allethrin increased acetylcholine levels in the hippocampus.	*Sprague Dawley rats*	[59]
	Bifenthrin, 0.6 and 2.1 mg/kg-BW	Bifenthrin treatment significantly reduced Na^+^/K^+^-ATPase and Mg^2+^-ATPase activities in the hippocampus, decreased mRNA expression and protein levels of Nurr-1, and lowered AChE and BuChE activities.	*Wistar Rats*	[95]
	Allethrin, (10, 25, 50, 100, 200) μM	Allethrin reduced cell viability in human dopaminergic neuroblastoma SH-SY5Y cells, and elevated ROS levels.	*Human*	[96]
Cardiac toxicity	Organophosphate, unclear dose	QT prolongation, resulting in torsade de pointes (TdP) and myocardial damage.	*Human*	[67,68]
	Dichlorvos, 170 μM	Dichlorvos caused necrotic cell death in H9C2 cells by significantly increasing the levels of intracellular and mitochondrial ROS, thus triggering oxidative stress in cardiac cells.	*Wistar rats*	[71]
Kidney	Deltamethrin, 300 mg/kg diet; deltamethrin 1.28 mg/kg-BW	Deltamethrin increased ALT, AST, urea, and creatinine in the serum of treated birds. It also led to severe kidney damage, evidenced by glomerular hyperplasia, necrosis, tubular dilation, epithelial cell sloughing, and the infiltration of lymphocytes.	*Cobb broiler chicks*, *Sprague Dawley rats*	[79,94]
	Triflumuron, 500 mg/kg-BW	Increment in lipid peroxidation and antioxidant enzyme activity, as well as the deterioration of proteins.	*Balb/C mice*	[76]

BW, body weight.

## 3. Effect of Pesticides on Reproductive Functions

### 3.1. General Reproductive Toxicity

Exposure to endocrine-disrupting chemicals can result in various reproductive toxicities in both females and males, affecting both humans and animals. These chemicals have the potential to disrupt the delicate balance of the reproductive system, as evidenced by changes in hormone levels in the blood, irregularities in the ovarian cycle, follicular damage, and reduced fertility [43,97]. Organochlorine compounds such as methoxychlor, chlordecone, dieldrin, docofol, endosulfan, dichlorvos, and permethrin have been reported to disrupt the estrus cycle in rats [98]. Furthermore, pesticides have been found to decrease the number of corpora lutea, pivotal in secreting progesterone to maintain the uterine lining during the menstrual cycle and supporting early pregnancy (Table 2) [98]. Cypermethrin and methamidophos, for instance, induce the deterioration of corpus luteal cells [99]. Moreover, certain pesticides, including fipronil, DDT, endosulfan, dieldrin, and other organophosphates, possess estrogenic potential, which may suppress the release of hypothalamic gonadotropin-releasing hormone (GnRH) and impair the synthesis and secretion of follicle-stimulating hormone (FSH) and luteinizing hormone (LH) by the pituitary gland through a negative-feedback mechanism [40,43,97,98,100]. Organophosphates possess the ability to disrupt the endocrine system by directly interacting with receptors or affecting the enzymes responsible for synthesizing and metabolizing steroid hormones [40,51,100]. Pesticides can function as hormonal disruptors in both males and females. For instance, 3,5,6-trichloro-2-pyridinol (TCPY), a metabolite of CPF and chlorpyrifos-methyl, can inhibit testosterone activity through negative feedback [101,102]. Similarly, fipronil can exert long-term effects on the epididymis and result in a reduction in testosterone secretion, highlighting the association of pesticides with altered endocrine function of the hypothalamic–pituitary–gonadal axis [101,103]. The androgen receptor (AR) is a crucial transcription factor present in the testes. When androgens bind to the AR, they activate it, playing a significant role in the development of male sex organs and the maintenance of male reproductive functions [104,105]. However, pyrethroid pesticides such as cyfluthrin, cyhalothrin, cypermethrin, deltamethrin, fenvalerate, permethrin, and 3-phenoxybenzoic acid (3-PBA) have been observed to exhibit antagonistic effects on the androgen receptor in MDA-kb2 cells, suggesting that they might induce prostate and testicular cancers [106]. Wang et al. found that beta-cyfluthrin induces the swelling and degeneration of Leydig cell mitochondria and the smooth endoplasmic reticulum, resulting in the formation of concentric circles. Conversely, lambda-cyhalothrin reduces seminal vesicle weight, epididymal sperm count, and motile spermatozoa while increasing the number of morphologically abnormal spermatozoa [104]. Another study indicated that cypermethrin disrupts the IL-6-mediated activation of AR via the Janus kinase (JAK)/transcription 3 (STAT3) signaling pathway, potentially influencing male reproductive function [107]. Additionally, exposure to organophosphate has been linked to various issues with semen quality, such as lower sperm count, reduced motility and viability, decreased density, and heightened DNA damage and abnormal sperm shape [108]. Moreira et al. 2021 found that the exposure of Leydig cells to an organophosphate insecticide reduced serum levels of testosterone while increasing the levels of LH and FSH, and it also diminished the size of Leydig cells. Moreover, Sertoli cells, which provide nutritional support to developing germ cells by producing lactate from glucose, also play a crucial role in maintaining the blood–testis barrier (BTB). However, methyl parathion and organophosphorus pesticides disrupt BTB integrity and reduce sperm quality due to oxidative stress (Table 2) [109]. Therefore, pesticides have been demonstrated to disrupt reproductive physiology and organ function, posing significant risks to reproductive health. 

### 3.2. Effect of Pesticides on Male Fertility

**Figure 1:**
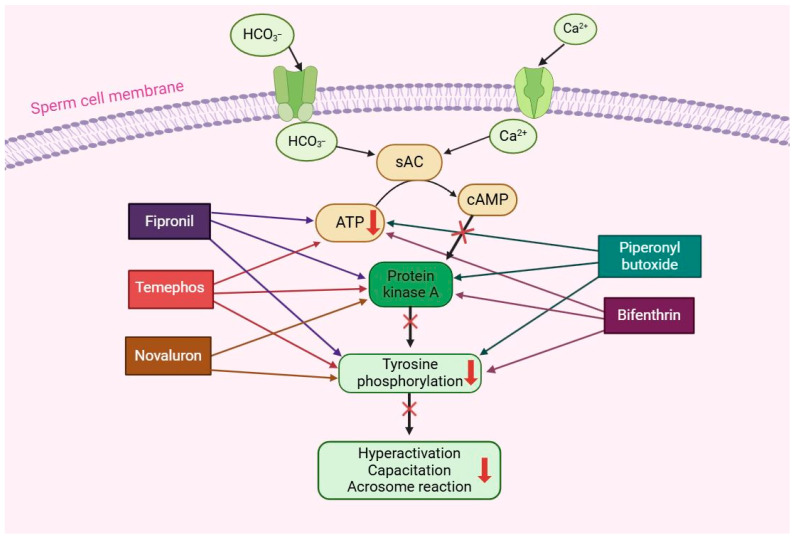
Effect of pesticide exposure on sperm capacitation. Pesticides interrupt sperm capacitation through the disruption of protein kinase A (PKA), tyrosine phosphorylation, ATP, cAMP, and sperm hyperactivation. Each pesticide has different arrow color which indicate that pesticide has effect on ATP, PKA, and Tyrosine phosphorylation, thus, decreasing sperm hyperactivation, capacitation, and acrosome reaction.

Male fertility refers to the ability of male animals or humans to successfully impregnate a female. It can be influenced by various factors, including environmental, genetic, and lifestyle conditions [118]. Pesticides have been identified as factors that can result in reduced male fertility due to their toxicity in male reproductive organs and germ cells [119]. Reproductive organs play a crucial role in fertility as they provide the necessary environment for sperm cell proliferation and differentiation [120,121]. Sperm cells undergo capacitation as they traverse the female genital tract. During this process, sperm cells undergo alterations in motility patterns, intracellular Ca^2+^ and pHi levels, the distribution of membrane lipids and proteins, and the activation of signaling pathways such as cyclic adenosine monophosphate (cAMP), PI3K/AKT, PKA, and tyrosine phosphorylation, thereby enabling them to penetrate and fertilize an egg [28,30,122]. Spermatozoa encounter elevated levels of Ca^2+^ and HCO_3_^−^ in the female genital tract, resulting in cholesterol efflux and an influx of calcium ions and HCO_3_^−^ through the sperm plasma membrane [123]. The increase in intracellular Ca^2+^ and HCO_3_^−^ levels modulates the activity of soluble adenylyl cyclase (sAC), leading to the elevation of intracellular cAMP levels and the activation of protein kinase A, subsequently inducing tyrosine phosphorylation and sperm hyperactivation, thereby enhancing the acrosome reaction [26,31,122,124]. The disruption of sperm capacitation can lead to male infertility, a concern that has been exacerbated by increased exposure to pesticides [28,119]. For instance, studies have shown that novaluron, a benzoylphenylurea insecticide, affects sperm capacitation by reducing sperm motility patterns and motion kinematics parameters, suppressing the acrosome reaction through alterations in PKA and tyrosine phosphorylation (Figure 1) [25]. Additionally, permethrin and cypermethrin, categorized as pyrethroid insecticides, have been found to cause a progressive decrease in sperm motility and motion kinematic parameters [122]. Various pesticides, including temephos, flufenoxuron, bifenthrin, and fipronil, have been identified as negatively impacting sperm motility and motion kinematics by reducing the levels of intracellular ATP, which serves as the energy source for sperm movement (Figure 1) [26,27,28,33]. Bifenthrin, for example, abnormally elevates PKA activation by inhibiting PKA-mediated signaling, consequently altering tyrosine phosphorylation and leading to a poor acrosome reaction [27]. Furthermore, piperonyl butoxide, a synergist pesticide, decreases the levels of PKA substrates during sperm capacitation (Figure 1) [31]. This adversely affects sperm motility and motion kinematics, resulting in the inability of sperm cells to undergo the capacitation process and ultimately causing male infertility. The PI3K/AKT signaling pathway plays an important role in the sperm maturation process as it affects the increase in intracellular calcium levels and the phosphorylation of proteins present in sperm flagella, thereby regulating flagella movement [125]. Studies have reported that CPF regulates PI3K/AKT signaling in Leydig cells and TM4 cells, leading to apoptosis [126]. It can also disrupt the cell cycle in porcine ST cells [127]. However, there is a scarcity of research on the effects of pesticides on the PI3K/AKT signaling pathway during sperm capacitation. While the exact molecular mechanisms by which pesticides affect sperm capacitation are not fully understood, it is evident that they have a detrimental effect on sperm function, ultimately resulting in male infertility.

## 4. Summary and Future Prospects

Currently, pesticides are extensively used worldwide across various industries, including agriculture and healthcare. This widespread application increases the risk of exposure to pesticides for both humans and animals, thereby posing significant health risks. Pesticide exposure has been associated with a range of health ailments, including cardiac toxicity, neurotoxicity, hepatotoxicity, and pulmonary toxicity, as well as various effects on the skin and eyes. Additionally, pesticides have been strongly linked to reproductive issues, particularly male infertility. Researchers have identified mechanisms through which pesticides mimic hormones, inducing adverse health effects. However, there is a lack of studies elucidating the molecular mechanisms through which pesticides influence male reproduction, specifically sperm capacitation. While some pesticides have been reported to affect the PI3K/AKT pathway in sperm, further research is needed to elucidate the underlying mechanisms at a more detailed molecular level. While this review provides insights into the health risks of pesticide exposure in terms of male reproductive function, limitations exist due to the scope of the existing literature and variability in study methodologies. Furthermore, these implications underscore the critical need for prioritizing health education, research initiatives, and regulatory measures to effectively mitigate the adverse effects of pesticides on male reproductive health. Understanding these mechanisms is essential to raise awareness about the health risks of pesticide exposure and to encourage their cautious use.

## Figures and Tables

**Table 2:** Pesticides as endocrine disruptors in the reproductive system and their toxic effects.

Pesticide, Dose	Effect	Species	Reference
Chlorpyrifos, 7.45 mg/kg-BW	- Reduced testicular weight and induced testicular tissue damage. - Vacuolar degenerative changes both in Sertori and Leydig cells.	*Albino rats*	[110]
Iprodione, 200 mg/kg-BW and their mixtures			
Imidacloprid, 5 and 10 mg/kg	- Generation of free radicals in testicular tissues, potentially leading to oxidative stress. - The thickness of the tunica albuginea was greatly reduced, and spermatogenic cells detached from the basement membrane, showing an irregular arrangement. Additionally, there was a reduction in the Leydig cell population.	*Dawley rats*	[111]
Imidacloprid, (0.5 mL (100 mg)/L was sprayed on green grass in the field)		*Rabbit*	[112]
Atrazine, 200 mg/kg-BW	- Significantly reduced epididymis weight and increased catalase activity, which may result in the conversion of H_2_O_2_ into hydroxyl radicals, leading to oxidative stress. - Reduced sperm count. - Decreased testis and epididymis weight and significantly reduced the number of sperm in epididymis and testes. In addition, it reduced sperm motility and serum testosterone levels.	*Wistar rats*	[113]
Atrazine, 120 mg/kg-BW		*Sprague Dawley rats*	[114]
Malathion, 10 or 50 mg/kg-BW	- Significantly reduced sperm number and daily sperm production in testis and increased the number of abnormal spermatozoa.	*Wistar rats*	[115]
Mixture of dichlorvos (2.3), dicofol (2.1), dieldrin (0.05), endosulfan (3.8), and permethrin (25) (mg/kg)	- Due to estrogenic activity resulting in decreased levels of GnRH, FSH, and LH hormones, animals exhibited a reduced number of follicles and corpora lutea. - Resulted in shorter proestrus and diestrus and longer metestrus in Wistar rats at a high dose, whereas Lewis rats generally showed a short estrus cycle at a low dose.	*Sprague Dawley*, *Wistar*, *and Lewis rats*	[98]
Cypermethrin (10 ppm) and methamidophos (10 ppm)	- Deterioration of corpus luteal cells in a concentration-dependent manner. - Decreased sperm count, and inhibited the development of Leydig cells in late puberty, decreased testosterone, and caused significant elevation in serum LH at 50 mg/kg-BW. It also increased the ROS level in Leydig cells at 200 μM.	*Bovine*	[99]
Cypermethrin, 50 mg/kg-BW/day and 200 μM		*Sprague Dawley rats*	[116]
Fipronil, 5 mg/kg-BW	- Exhibited edema around the seminiferous tubules, degeneration at different spermatogenesis stages, and a reduced number of sperm in the tubule lumens.	*Albino rats*	[117]
Deltamethrin, 0.6 mg/kg-BW	- Reduced blood testosterone levels.	*Mice*	[102]
